# An immunohistochemical, enzymatic, and behavioral study of CD157/BST-1 as a neuroregulator

**DOI:** 10.1186/s12868-017-0350-7

**Published:** 2017-03-24

**Authors:** Haruhiro Higashida, Mingkun Liang, Toru Yoshihara, Shirin Akther, Azam Fakhrul, Cherepanov Stanislav, Tae-Sik Nam, Uh-Hyun Kim, Satoka Kasai, Tomoko Nishimura, Naila Al Mahmuda, Shigeru Yokoyama, Katsuhiko Ishihara, Maria Gerasimenko, Alla Salmina, Jing Zhong, Takahiro Tsuji, Chiharu Tsuji, Olga Lopatina

**Affiliations:** 1 Research Centre for Child Mental Development, Kanazawa University, Kanazawa, 920-8640 Japan; 2 Department of Biochemistry, Chonbuk National University Medical School, Jeonju, South Korea; 3 Department of Immunology and Molecular Genetics, Kawasaki Medical School, Kurashiki, Okayama 701-0192 Japan; 4 Department of Biochemistry, Medical, Pharmaceutical and Toxicological Chemistry, Krasnoyarsk State Medical University, Krasnoyarsk, Russia 660022

## Abstract

**Background:**

Recent rodent and human studies provide evidence in support of the fact that CD157, well known as bone marrow stromal cell antigen-1 (BST-1) and a risk factor in Parkinson’s disease, also meaningfully acts in the brain as a neuroregulator and affects social behaviors. It has been shown that social behaviors are impaired in *CD157* knockout mice without severe motor dysfunction and that *CD157/BST1* gene single nucleotide polymorphisms are associated with autism spectrum disorder in humans. However, it is still necessary to determine how this molecule contributes to the brain’s physiological and pathophysiological functions.

**Methods:**

To gain fresh insights about the relationship between the presence of CD157 in the brain and its enzymatic activity, and aberrant social behavior, *CD157* knockout mice of various ages were tested.

**Results:**

CD157 immunoreactivity colocalized with nestin-positive cells and elements in the ventricular zones in E17 embryos. Brain *CD157* mRNA levels were high in neonates but low in adults. Weak but distinct immunoreactivity was detected in several areas in the adult brain, including the amygdala. CD157 has little or no base exchange activity, but some ADP-ribosyl cyclase activity, indicating that CD157 formed cyclic ADP-ribose but much less nicotinic acid adenine dinucleotide phosphate, with both mobilizing Ca^2+^ from intracellular Ca^2+^ pools. Social avoidance in *CD157* knockout mice was rescued by a single intraperitoneal injection of oxytocin.

**Conclusions:**

CD157 may play a role in the embryonic and adult nervous systems. The functional features of CD157 can be explained in part through the production of cyclic ADP-ribose rather than nicotinic acid adenine dinucleotide phosphate. Further experiments are required to elucidate how the embryonic expression of CD157 in neural stem cells contributes to behaviors in adults or to psychiatric symptoms.

**Electronic supplementary material:**

The online version of this article (doi:10.1186/s12868-017-0350-7) contains supplementary material, which is available to authorized users.

## Background

Accumulating evidence suggests that immune-related molecules tend to be involved in neuronal development and maintenance, and a number of molecules have been reported to possess neuronal functions, such as complement C4 [1], C1q [2], schnurri-2 [3], and CD38 [4]. CD38, originally identified as playing a role in hematopoietic cells and leukocytes and in the pathogenesis of leukemia and HIV infection [5–8], has a critical role in oxytocin (OT) secretion in the hypothalamus and in the regulation of social memory and social interactions [4, 9, 10]. The mechanism underlying the regulation of OT release from oxytocinergic neurons involves increases in intracellular Ca^2+^ concentrations by mobilization of Ca^2+^ from ryanodine receptors [11–13] to intracellular Ca^2+^ pools by cyclic ADP-ribose (cADPR). CD38, together with CD157, is a member of the ADP-ribosyl cyclase family, which catalyzes the formation of cADPR from β-NAD^+^ [14, 15].

CD157, discovered as bone marrow stromal cell antigen-1 (BST-1) by Hirano and colleagues [14–16], is a cell-surface molecule that supports pre-B cell growth with enhanced expression on bone marrow stromal cell lines derived from rheumatoid arthritis patients [17]. BST-1, expressed by myeloid cells as a molecule capable of signal transduction, was clustered as CD157 after gene cloning [14, 17]. CD157 is expressed abundantly in the immune tissues of adult mice, *e.g.*, in the spleen [5, 7, 13]; therefore, CD157 can be classified as an *immune* molecule. Under inflammatory conditions, the homophilic binding of CD157 between the blood vessel endothelial cells and lymphocytes promotes lymphocyte migration [5, 13]. CD157 is considered to play a role in the progression of chronic leukocytic leukemia [7]. In addition, CD157 is involved in the pathophysiology of various diseases [7, 12]. Its involvement in rheumatoid arthritis was implicated in the study that reported its discovery [17]. CD157 has been shown to support B lymphocyte survival in rheumatoid arthritis, although the mechanistic basis of this observation is not yet clear. CD157 stimulates neutrophil migration to sites of inflammation [5, 13, 15]. The expression of CD157 in ovarian carcinoma cells is an indicator of malignancy and a higher rate of metastasis [18].

While investigations have intensively focused on inflammation and the immune system, little information regarding the other functions of CD157 has been reported. In 2009, however, genome-wide association studies (GWASs) identified single nucleotide polymorphisms (SNPs) in the *CD157/BST1* gene on human chromosome 4p15 as new risk factors for Parkinson’s disease (PD) in the Japanese population [19]. Fifteen subsequent reports confirmed the above findings in different populations, including subjects of European descent from France, Australia, the UK, and the Netherlands, and in Ashkenazi Jews and Asian populations, such as the Chinese and Korean populations [20–35]. Although six reports yielded contradictory findings [36–41], all other GWASs identified intronic SNPs in the *CD157/BST1* gene that predispose carriers to PD. These GWASs indicated that *CD157* SNPs confer a small (usually 1.1–1.3-fold increase) risk for PD. Thus, as suggested by a previous study in Taiwan [40], additional genetic and environmental factors may be needed to determine the pathogenic role of CD157. Nonetheless, it is worth considering how CD157 contributes to the occurrence of PD or to at least one or a variety of PD symptoms. Previously, we examined the neuronal function of CD157 by analyzing the phenotypes of *CD157* deletion mice that would presumably provide insights into the neuronal functions of CD157 [42]. Interestingly, the results obtained indicated no motor dysfunction but social behavioral impairments in *CD157* knockout (KO, *CD157* ^−*/*−^) mice such as apathy-, anxiety-, and depression-like behaviors [42], which are slightly different from the expected phenotypes involving motor dysfunction that are commonly observed in PD.

Recently, Wu et al. reported that nanodiamond particles can be transferred into transplanted CD45^−^CD54^+^CD157^+^ lung stem/progenitor cells [43]. Yilmaz et al. reported that fasting results in the activation of the mTORC1 pathway and induction of CD157 in Paneth cells in the adult mouse intestine [44, 45]. The product of the CD157 catalyzed reaction, cADPR, inhibits differentiation of intestinal stem cells to acinar cells, and thus facilitates proliferation of intestinal stem cells. These results suggest that CD157 is an *enteric* protein and that CD157 plays a role in the stem cells of two organs. However, the question about the presence of CD157 in neuronal stem cells remains unanswered.

Here, we present new data to evaluate our previous findings regarding the functional roles of CD157 in the nervous system. We also present results with CD38 for comparison. Finally, we discuss the limitations of our results and possibilities about the role of CD157 in the progression of PD in relation to psychiatric symptoms as well as other psychological disorders.

## Results

**Fig. 1:**
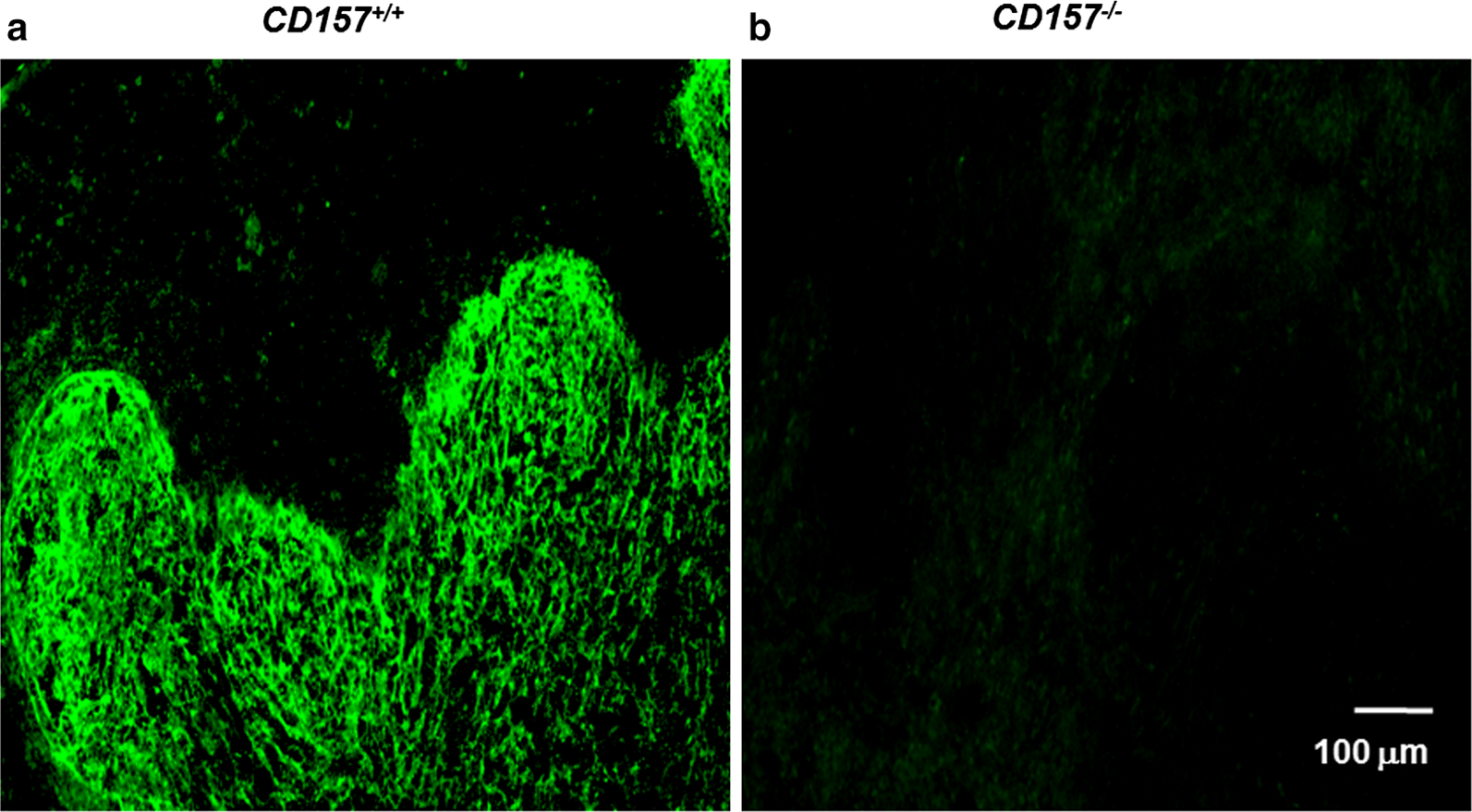
Specificity of rabbit antiserum for murine CD157. Representative images of CD157 immunoreactivity (*green*) in the spleens of wild-type (*CD157* ^+*/*+^; **a**) and *CD157* KO (*CD157* ^−*/*−^; **b**) adult male mice. The tissues were incubated with the rabbit antiserum against murine CD157. After washing, the sections were treated with Alexa Fluor 488

We used polyclonal antiserum raised in rabbits against a chimeric fusion protein of murine CD157 that had been preabsorbed with CD38, to examine its specificity for CD157. In C57BL/6N wild-type adult mice, CD157 immunoreactivity was shown in the white pulp region of the spleen, where CD157 was enriched (*CD157* ^+*/*+^, Fig. 1), while no staining was observed in *CD157* knockout (*CD157* ^−*/*−^) mice. These immunofluorescence results clearly indicate that our antibody is specific to mouse CD157. On the basis of this specificity, we decided to assess CD157 immunoreactivities in the brains of embryos.

### Presence of CD157 in brain stem cells

**Fig. 2:**
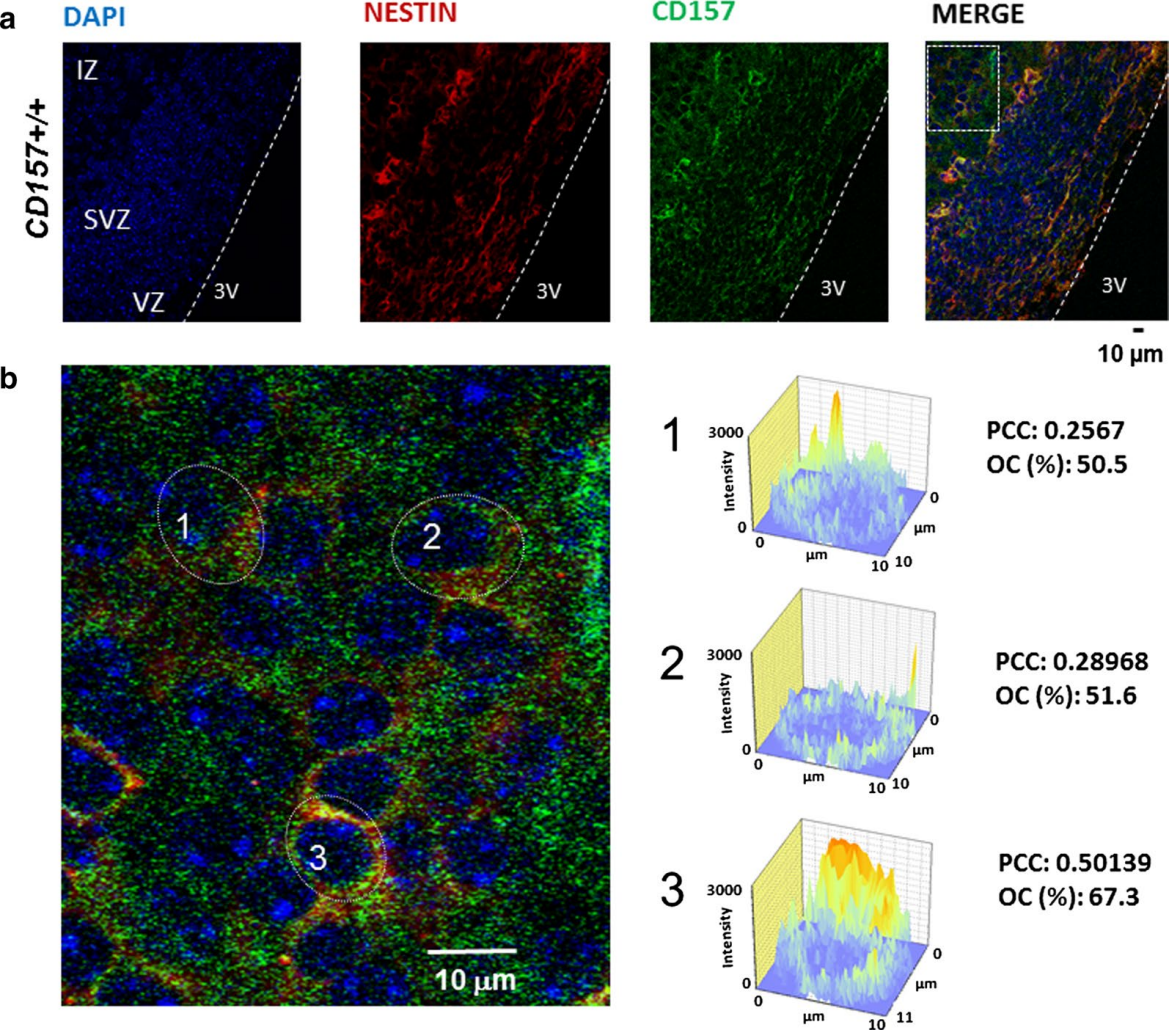
Immunohistochemical analysis of CD157 expression in the embryonic brain. **a** Representative images from the E17 embryonic hypothalamus: CD157 stained in *green*, nestin in *red*, and the nucleus in *blue*. **b** An enlarged image indicated in (**a**) (*a square enclosed by a dotted line*) shows the colocalization patterns of CD157 (*green*) and nestin (*red*) in individual cells. Colocalization diagrams of *green* (CD157) and *red* (nestin) channels in three cells indicated in the image illustrate overlap in the individual cells. The *X*- and *Y*-axes indicate cell size in µm; the *Z*-axis-fluorescence signal intensity. Colocalization is estimated by calculating Pearson’s correlation coefficient (PCC) and overlap coefficient (OC). Intermediate zone (IZ), subventricular zone (SVZ), ventricular zone (VZ), and third ventricle (3V)

Strong staining for CD157 in immunofluorescence images obtained using a confocal microscope was seen in the hypothalamus of E17 embryos (Fig. 2). CD157 immunoreactivity was detected in the cytoplasm or at the cell surface of many but not all nestin-positive cells in the ventricular and subventricular zones beside the third ventricle (Fig. 2a). This colocalization was examined further by quantitative colocalization analysis (46). As shown in Fig. 2b, three representative cells show that the degree of overlap of the fluorescence signals is greater than 50%. We estimate that this colocalization is limited to the embryonic stem cells. However, we did not examine the presence of CD157 in adult mouse brain stem cells, although this is an interesting topic and should be examined further.

### mRNA expression in neonates and adult male mice

**Fig. 3:**
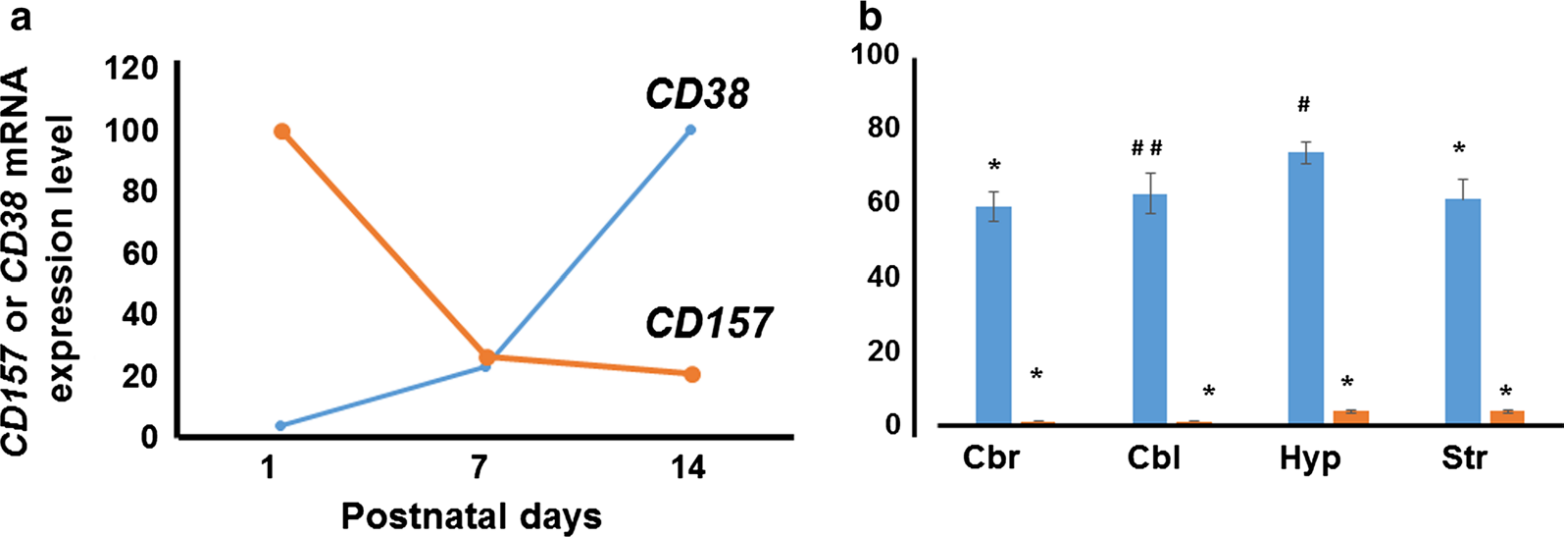
*CD157* and *CD38* mRNA expression in the brain. **a** Schematic drawing of the time course of mRNA expression in the whole brains of pups on postnatal days 1, 7, and 14. One hundred percent refers to values on postnatal days 1 or 14 for *CD157* and *CD38*, respectively. Data for *CD157* and *CD38* were obtained from previous reports by Lopatina et al. [42] and Higashida et al. [45], respectively (*n* = 5 mice for *CD157* and *n* = 4 mice for *CD38*). **b** Quantitative data obtained by RT-PCR analysis of *CD157* and *CD38* mRNA expression in four brain regions of adult male mice using β-actin mRNA as an internal control. Values show percentages of means  ±  S.E.M. (*n*  =  5 independent experiments) of *CD157*/actin with values in the spleen set to 100. One-way ANOVA reveals *F*_2,40_ = 195.94, *P* < 0.0001 for *CD157* and *P* < 0.0001, *F*_4,20_ = 9.78 for *CD38*. Significant from density of spleen at *P < 0.0001, ^##^ *P* < 0.001, ^#^ *P* < 0.05, respectively. *Cbr* cerebrum, *Cbl* cerebellum, *Hyp* hypothalamus, *Str* striatum

In support of the above observation and as reported previously [42], the brain *CD157* mRNA levels decreased markedly 7–14 days postnatally from the relatively high levels in the embryonic period (shown in Fig. 3a (*n* = 5 mice)). This time course is the opposite of that of *CD38*, whose mRNA levels on postnatal day 14 represent a >25-fold increase from that on postnatal day 1 (*n* = 4 mice) [45].

The levels of *CD157* mRNA expression in different brain regions (cerebrum, cerebellum, hypothalamus, and striatum) in adult male C57BL/6 mice were extremely low compared to that in the spleen (*P* < 0.001, *n*  =  5) (One-way ANOVA, *F*_2,40_  =  195.94, *P*  <  0.0001; Fig. 3b; Additional File 1). In marked contrast, *CD38* mRNA was expressed abundantly in the four brain regions examined, with no significant difference in density among regions, although these densities were significantly lower than that in the spleen (*P*  <  0.05 for the hypothalamus; *P*  <  0.001 for the cerebellum and striatum; *P*  <  0.0001 for the cerebrum: One-way ANOVA, *P*  <  0.0001, *F*_4,20_  =  9.78, *n* = 4). Taken together, these mRNA and protein expression studies show the quite distinct expression profiles of CD38 and CD157. However, for a more quantitative comparison, it is necessary to analyze brain samples by using the SYBR–Green I-based reverse transcription-quantitative polymerase chain reaction assay.

### Immunohistochemistry in the adult brain

**Fig. 4:**
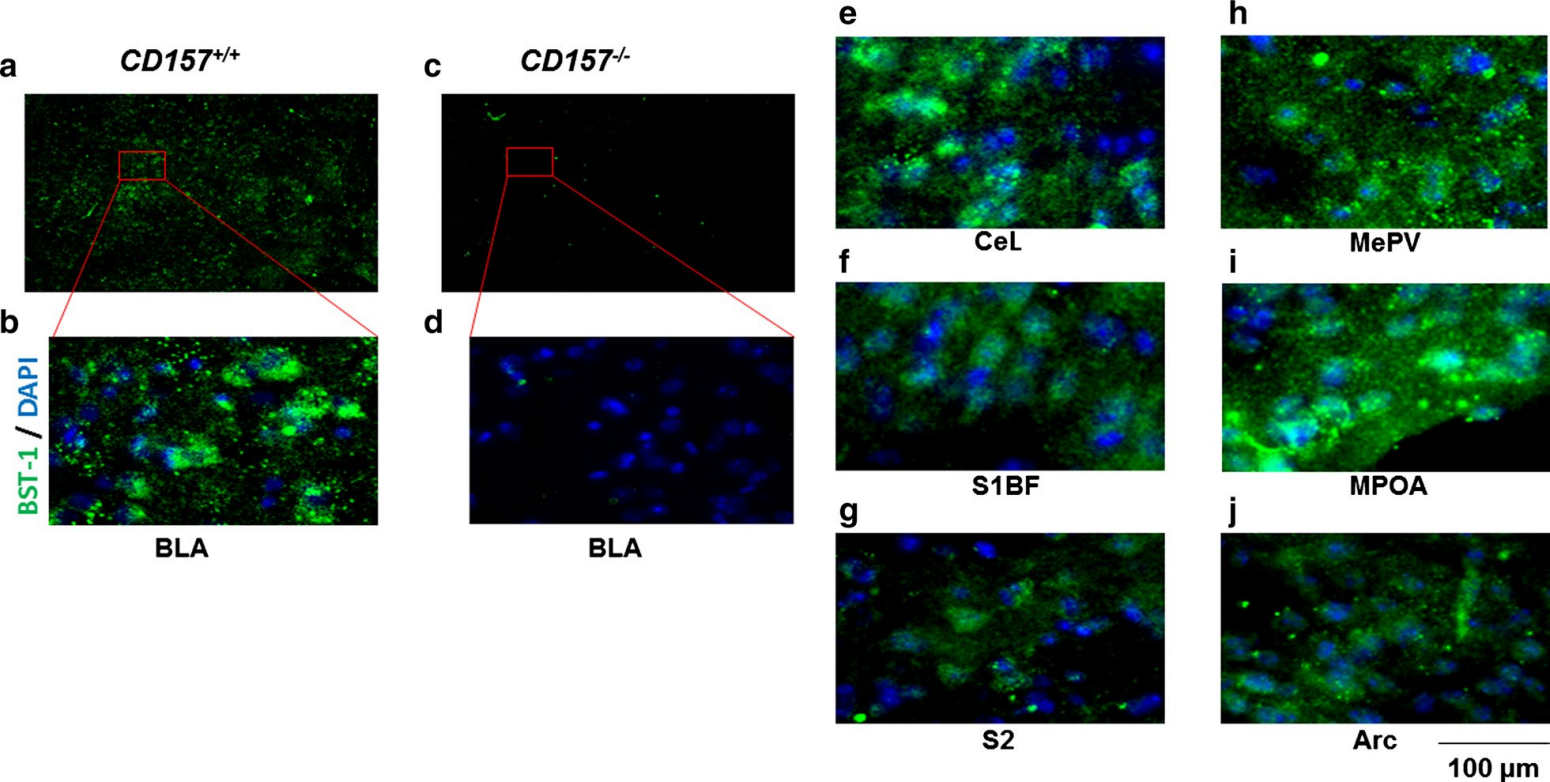
Immunohistochemical analysis of CD157 expression in the brains of adult male mice. Frozen sections of wild-type (*CD157* ^+/+^) and knockout (*CD157* ^−*/*−^) mice were incubated with rabbit antiserum against murine CD157. After washing, the sections were treated with Alexa Fluor 488. Representative photographs show CD157 (*green*) and Hoechst-stained nuclei (*DAPI, blur*) immunoreactivity in *CD157* ^+/+^ **a,b, e**–**j** and *CD157* ^−*/*−^ **c**, **d** mouse brains. The *insets* are enlarged areas indicated in the lower magnification photographs. Brain areas examined are: **a**–**d** basolateral amygdaloid nucleus, anterior part (BLA); **e** central amygdaloid nucleus (CeL); **f** primary somatosensory cortex (S1BF); **g** secondary somatosensory cortex (S2); **h** medial amygdaloid nucleus, posteroventral part (MePV); **i** medial preoptic area (MPOA); **j** arcuate hypothalamic nucleus (Arc). The *scale bar* represents 100 μm

The expression of CD157/BST-1 protein in the brains of adult male mice was examined using our polyclonal antiserum [42]. Weak but distinct immunoreactivity was detected in the amygdala (basolateral amygdaloid nucleus, anterior part (BLA; Fig. 4a, b)) of *CD157* ^+/+^ mouse, but identical immunoreactivities were not found in *CD157* ^−*/*−^ mice (Fig. 4c, d). CD157 immunoreactivity was detected in the central amygdaloid nucleus (CeL; Fig. 4e), medial amygdaloid nucleus, posteroventral part (MePV, Fig. 4h), somatosensory cortex (primary somatosensory cortex, barrel field (S1BF; Fig. 4f)), medial preoptic area (MPOA; Fig. 4i), secondary somatosensory cortex (S2; Fig. 4g), and arcuate hypothalamic nucleus (Arc; Fig. 4j) of the *CD157* ^+/+^ mouse brain. Little or no immunoreactivity was detected in the other brain regions examined: the hippocampus, piriform cortex, perirhinal cortex, retrosplenial granular cortex c region or the nucleus accumbens. This expression pattern of CD157 corresponded well with our previous findings [42], although further examinations may be necessary because the volume of the amygdala was smaller in KO mice than in control mice [42].

### Enzymatic activity of CD157

An additional issue to be resolved is whether the functions of CD157 in neural stem cells as well as brain cells are mediated by the enzymatic products of CD157, cADPR and nicotinic acid adenine dinucleotide phosphate (NAADP), which mediate Ca^2+^ mobilization from Ca^2+^ pools [12, 46]. This was originally reported in bone marrow mesenchymal stem cells by Tao et al. [47], who showed that cADPR is a novel regulator of Ca^2+^ oscillations in human mesenchymal stem cells. They reported that cADPR permeates the cell membrane through nucleoside transporters and increases Ca^2+^ oscillations by the activation of another molecule, the TRPM2 cation channel, resulting in enhanced phosphorylation of ERK1/2, and thereby stimulating human mesenchymal stem cell proliferation.

CD157 and CD38 belong to a family with ADP-ribosyl cyclase activity. The ADP-ribosyl cyclase activity of CD157 is weaker than that of CD38 [6]. However, it is not clear whether CD157 has other enzyme activities, such as NAD glycohydrolase or NAD base exchange activities. The product of base exchange is NAADP [6, 12]. NAADP also has Ca^2+^ mobilization activity from different Ca^2+^ pools [12, 48].

**Fig. 5:**
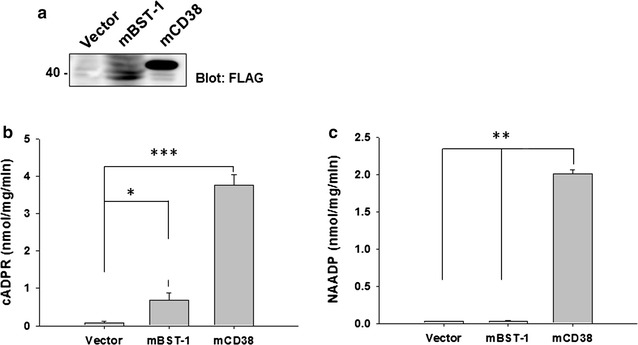
Enzymatic synthesis of cADPR and NAADP by FLAG-fusion proteins (mBST-1 and mCD38). **a** Western blot analysis of FLAG-fusion proteins from cell lysates of vector (50 µg), mouse BST-1 (mBST-1, 50 µg), and mouse CD38 (mCD38, 10 µg) using anti-FLAG antibody. **b**, **c** Enzyme activities for cADPR and NAADP synthesis were measured by incubation of purified FLAG-fusion proteins (vector, mBST-1 or mCD38) with appropriate substrates as described in the “Methods” section. *P < 0.001, **P < 0.0005, ***P < 0.0001. The mean ± S.E.M. of three independent experiments is shown

We examined whether CD157 has two enzyme activities, and especially its base exchange activity, by independently transfecting HEK cells with mouse *CD157* or *CD38* genes. The representative western blot analysis shows protein bands of expressed CD157 and CD38 (Fig. 5a). First, the ADP-ribosyl cyclase activity of *CD157*-transfected cells was higher than that of vector-transfected control cells (*P*  <  0.001) and lower than that of *CD38*-transfected cells (*P*  <  0.0001; *F*_8,34_ = 9.01, *n* = 3, one-way ANOVA; Fig. 5b; Additional File 2). Figure 5c shows that CD157 has little or no base exchange activity (*F*_2,7_  =  36.26, *P*  <  0.0001, *n* = 3): *CD157*-transfected cells showed no or little NAADP synthetic activity, with no discernable differences from vector-transfected control cells. In sharp contrast, *CD38*-transfected cells possessed significantly higher NAADP synthetic activity (*P*  <  0.0001) compared to control cells (*F*_2,7_  =  36.26, *n* = 3; Fig. 5c; Additional File 2). These results suggested that the functional role of CD157 likely stems from the production of cADPR rather than NAADP. It was also confirmed that cADPR production by CD157 is not high, unlike CD38.

### Social behavior in *CD157* or *CD38* KO mice

**Fig. 6:**
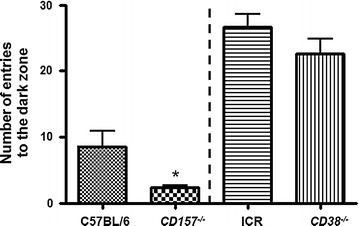
Behaviors of wild-type (C57BL/6 N for CD157 and ICR for CD38) or *CD157* and *CD38* knockout mice in the light–dark transition test. Values are transition numbers between the two compartments (light to dark zones). The results are expressed as the mean ± S.E.M., *n* = 5–10, *P < 0.05 compared with wild-type controls (two-tailed Student’s *t* test)

Severe anxiety-like and depression-related behaviors were observed previously in *CD157* KO male mice in the light–dark transition test, a standard anxiety-related behavioral test [42]. The transition from a light to a dark arena was significantly shorter in the *CD157* KO mice compared with the controls (two-tailed Student’s *t* test, *P*  <  0.05; Fig. 6; Additional File 3), suggesting anxiety related to a novel environment. For comparison, we also performed the test in *CD38* KO mice. While the entry frequency was markedly different, probably because of the different backgrounds of the two strains, we found no behavioral differences between *CD38* KO and wild-type ICR mice (Fig. 6 left panel; Additional File 3). These observations suggested that anxiety related to a novel environment is a characteristic of *CD157* KO mice, but not *CD38* KO mice.

**Fig. 7:**
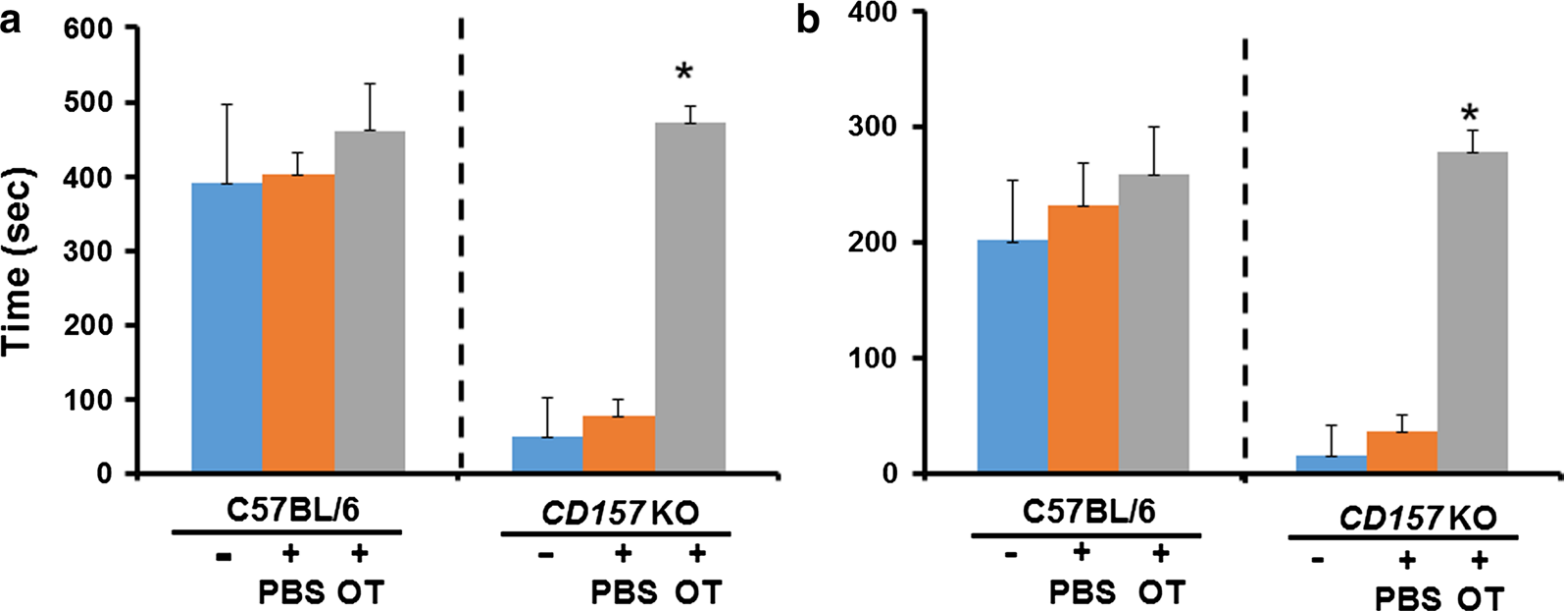
Social avoidance of adult male mice in the open field test. The test mouse was exposed to the open field for 20 min. A social (male mouse) target was present at the center of the inside arena. Time in the inside zone (**a**) and time spent immobile in the inside zone (**b**) were observed in wild-type (*blue*), *CD157* KO mice (*gray*), and *CD157* KO mice given an intraperitoneal injection of 100 ng/ml × 0.3 ml of OT (*orange*) are shown. Data are means  ±  S.D., *n*  =  8. Two-way ANOVA revealed significant *treatment* (PBS vs. OT) x *genotype* (wild-type vs. KO) interaction effects in **a** (*F*_2,42_ = 6.041, *P* = 0.0005) and **b** (*F*_2.42_ = 5.674, *P* = 0.0066). Values in *CD157* ^−*/*−^ mice treated with OT are significantly higher than those in *CD157* ^−*/*−^ mice with no treatment or those treated with PBS at *P*  <  0.001 in (**a**) and (**b**). *P < 0.001 compared OT treatment with no treatment or treated with PBS in *CD157* ^−*/*−^ mice (Tukey’s post hoc test)

Finally, the phenotype in the open field was examined in wild-type and *CD157* ^−*/*−^ mice. The time spent in the inside zone in an open field test was significantly shorter for *CD157* KO mice than wild-type controls (Fig. 7a). The interaction time with a social target mouse in the central arena was markedly shorter in *CD157* KO mice than in wild-type controls (Fig. 7b**)**, as judged from the duration of immobility. The immobility time was approximately half of the total time spent in the inside zone in both the KO and wild-type mice. Interestingly, this was rescued by a single intraperitoneal injection of OT with both time in the inside zone and immobility time increasing significantly (Fig. 7a, b; Additional File 4). The analysis of the results by two-way ANOVA revealed significant *treatment* (PBS vs. OT) x *genotype* (wild-type vs. KO) interaction effects (*F*_2,42_ = 6.041, *P* = 0.0005; Fig. 7a). Tukey’s multiple comparison tests detected significant differences in genotype (*F*_1,42_ = 23.08, *P* = 0.0001) and treatment (*F*_2,42_ = 11.06, *P* = 0.0001) effects. Two-way ANOVA detected significant *treatment* (PBS vs. OT) × *genotype* (wild-type vs. KO) interaction effects (*F*_2.42_ = 5.674, *P* = 0.0066; Fig. 7b). Tukey’s post hoc tests demonstrated significant differences in genotype (*F*_1,42_ = 17.49, *P* = 0.001) and treatment (*F*_2,42_ = 11.42, *P* = 0.0001) effects.

## Discussion

The most important result of the present study was the finding that the function of CD157 is mediated solely by the second messenger cADPR and not NAADP. This was the first clear demonstration of the differential action of CD157 in generating the Ca^2+^-related second messengers. CD38 is known to have a robust ability to catalyze cADPR as well as NAADP formation in several tissues, including the islets of Langerhans [4, 6, 8, 9, 12, 49]. NAADP, as a Ca^2+^ signaling messenger, plays an important role in insulin secretion [12]. However, this raises a critical question about CD157 mediating its functions in the nervous system and stem cells through cADPR but not NAADP formation. A good example is the crucial role of both cADPR and CD157 in intestinal Paneth cells [44, 45].

The second important finding was that CD157 immunoreactivity colocalized with nestin-positive cells and elements, probably neuroprogenitor or neurolineage cells, in the subventricular zone of embryos. Our results indicated that CD157 is a functional molecule, or at least is present in stem cells, as shown in the Paneth cells in the digestive tract [44, 45], lung [43], and mesenchymal cells [47]. The functional roles in these stem cells may be due to cADPR, as shown in Paneth cells. However, in the nervous system, CD157 may play a role in neuronal migration during neural stem cell proliferation and neurogenesis. It has been shown that CD157 binds with members of the integrin family [50]. Furthermore, integrin β3 and the serotonin transporter interact to modulate serotonin uptake in the mouse brain [51]. Therefore, it would be interesting to examine whether cADPR facilitates the self-renewal of neural stem cells and whether a tertiary complex of CD157, integrin, and the serotonin transporter is formed in the mouse brain.

**Fig. 8:**
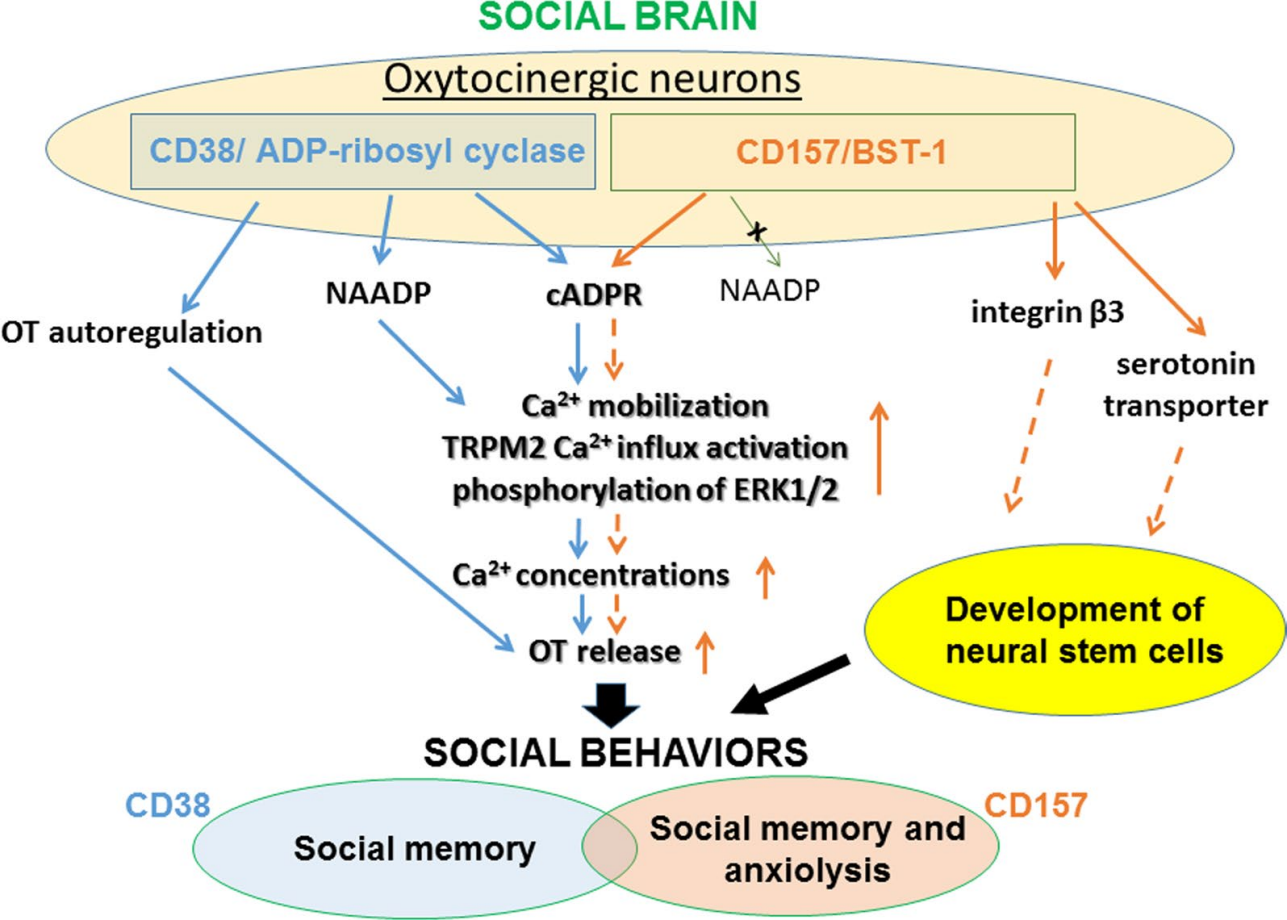
The scheme of CD38- and CD157-mediated molecular pathways in relation to social behavior. The scheme shows the possible molecular pathways of cADPR formation, OT release, and behavioral effects related to CD38 and CD157 in OT neurons in the social brain

CD157 is a sister molecule of the CD38 cell-surface antigen with ADP-ribosyl cyclase activity [5, 6, 12], but the phenotypes of *CD157* KO and *CD38* KO mice are quite different [4, 9–11, 46]. The role of CD38 in OT secretion into the brain has been established [4, 9–11]; CD38 mediates cADPR production, TRMP2 and ERK1/2 activation, Ca^2+^-mobilization, and OT release [47] (Fig. 8). Additionally, CD38 is involved in OT release by activating molecular cascades of OT autoregulation [4, 10]. In contrast, CD157 binds with the serotonin transporter and integrin β and invokes multiple circuits to control anxiety- and depression-like behaviors [13, 18, 42, 49]. CD157 plays a role in cADPR-induced OT release, which may not be identical to that of CD38. (Figure 8). The deficiency of CD157 leads to aberrant behaviors, such as increased anxiety. A decrease in volume of the amygdala, an important constituent of the “social brain,” might be caused by a loss of this molecule in the neural stem cells during the developmental stages [42].

The behavioral impairments in *CD157* KO mice were rescued by OT, probably because OT directly targets the intracellular signaling networks in the social brain downstream of CD157, which might be independent of CD38 and its downstream signaling networks (Fig. 8). This observation suggests that OT can be used for the treatment of social avoidance in psychological disorders. Furthermore, whether we can extend the results obtained in the mouse to human behavioral recovery is of interest, especially since there have been reports regarding the effectiveness of OT for impaired social interaction in cases of autism spectrum disorder (ASD) [53–55].

It has been shown that SNPs of *CD38* are associated with ASD [57–62]. As CD157 is abundant in the embryonic stages, speculation regarding an association between the SNPs of *CD157* and ASD is understandable. Recently, we found associations between ASD and three SNPs of *CD157* (rs4301112, rs28532698, and rs10001565). These SNPs have chromosomal locations (from chr4:15717226 to chr14:15722573) distinct from those associated with PD (chr14:15725766 to chr4:15737937) [63].

## Conclusions

The results of the present study indicate a novel neuronal role for CD157 in addition to its known functions in the digestive and immune systems. Therefore, CD157 can be referred to as a *neuro*-*entero*-*immunological* regulator [14]. The less abundant expression of CD157 in the adult brain raises questions about the common features associated with the late onset of PD. Because CD157 is expressed abundantly in the embryonic brain, it may be involved in the processes of neuronal development that relate to Psychiatric disorders, such as ASD and schizophrenia.

## Methods

### Animals

*CD157/BST1* KO and *CD38* KO mice were created as described previously [4, 15, 64]. Frozen *CD157* ^+/−^ fertilized oocytes were routinely inoculated into pseudopregnant foster mothers. Offspring were genotyped as described previously [15]. *CD157* and *CD38* KO mice were maintained by crossbreeding homozygous mutant mice. Slc:ICR (CD-10) outbred male mice (10–12 weeks old, 30–35 g body weight) were obtained from Japan SLC Inc. (Hamamatsu, Japan) via a local distributor (Sankyo Laboratory Service Corporation, Toyama, Japan). In over half of the experiments, the offspring of wild-type mice were bred in our laboratory colony, weaned at 25–30 days of age, and housed in same-sex groups of 3–5 animals. In general, 4–5 males were kept in one cage in the animal center under standard conditions (24 °C; 12/12-h light/dark cycle, with lights on at 8:45 a.m.) with food and water ad libitum. The breeding pairs were maintained separately (1 pair per cage). After parturition, the body weight of both male and female pups was measured daily in 10 pairs consisting of approximately 40 pups. At 21 days old, the offspring were removed for weaning and housed in same-sex sibling pairs. Subsequently, viability was assessed. All animal experiments were carried out in accordance with the Fundamental Guidelines for Proper Conduct of Animal Experiment and Related Activities in Academic Research Institutions under the jurisdiction of the Ministry of Education, Culture, Sports, Science and Technology of Japan and were approved by the Committee on Animal Experimentation of Kanazawa University.

### CD157 immunostaining

Embryos were removed from anesthetized dams. Adult male mice were anesthetized and perfused intracardially with cold phosphate-buffered saline (PBS) followed by cold 4% paraformaldehyde (PFA) in PBS. Their brains were removed and fixed in 4% PFA solution overnight at 4 °C. Sections were preincubated in blocking solution (3% bovine serum albumin and 0.3% Triton X-100 in PBS) for 1 h and then incubated overnight with an antibody against CD157. Anti-murine CD157 (BST-1) antiserum was prepared by immunizing rabbits with a chimeric fusion protein of murine CD157 and the Fc portion of human IgG1 (mBST-1Fc), and the reactivities to human IgG and murine CD38 were absorbed by human IgG Sepharose 6 and the transfectant, BAFmCD38, respectively, as described [42]. Goat anti-nestin antibody (sc-20978, 1:1000; Santa Cruz Biotechnology Inc., USA) was added to blocking solution. After three washes with washing buffer, the sections were incubated with goat anti-rabbit and donkey anti-goat IgG antibodies coupled to Alexa Fluor 488 and 594 (Invitrogen, Carlsbad, CA), respectively, and DAPI (Wako, Osaka, Japan) in blocking solution for 1 h at room temperature. Imaging was performed with an Olympus IX71 fluorescence microscope (Tokyo, Japan). We identified CD157-positive cells within a specific size range and above a constant threshold level of staining as follows: fluorescence diameter  <  15 μm and intensity >  50 (arbitrary units, a.u.) [65, 66]. The colocalization of CD157 and nestin immunoreactivities was estimated using Pearson’s correlation coefficient (PCC) and overlap coefficient (OC) with Olympus FluoView software (Ver.4.0a) [67].

### RT-PCR

Total RNA was isolated from mouse brain subregions using TRIzol Reagent (Invitrogen). cDNA was synthesized from 0.5 μg of total RNA using ReverTra Ace-α (Toyobo, Osaka, Japan) according to the manufacturer’s protocol. PCR was performed on a Mastercycler ep gradient S (Eppendorf, Hamburg, Germany) under the following conditions: 1 cycle at 94 °C for 30 s, followed by 25 or 30 cycles at 94 °C for 30 s, 58 °C for 30 s, and 72 °C for 40 s, with a final extension step at 72 °C for 1 min. The primer sequences used have been reported previously [15, 42]. RT-PCR products were separated electrophoretically on 1.2% gels and stained with ethidium bromide. Band intensity was measured from photographs using ImageJ. The data presented shows the ratio of *CD157* intensity to the intensity of β-actin in the same sample.

### Plasmids and transfection

Flag-mBST-1 and Flag-mCD38 cDNA were cloned from mouse spleen cDNA. mBST-1 and mCD38 were amplified by PCR using mBST-1-EcoR I forward (5′-CGGAATTCAATGGCTGTCCAGGGAGGCCT-3′), mBST-1-Xba I reverse (5′-GCTCTAGACGCCTGAGAACTTGAAGCCAAAG-3′), mCD38-EcoR I forward (5′-GGAATTCCATGGCTAACTATGAATTTAGCC-3′) and mCD38-Sal I reverse (5′-ACGCGTCGACGCGTATTAAGTCTACACGATGGG-3′) primers. The PCR products were ligated into a pFLAG-CMV2 expression vector. HEK293 cells were transfected with the plasmids using Lipofectamine 2000 transfection reagent (Invitrogen) according to the manufacturer’s instructions. Cells were used 24 h post-transfection.

### Measurement of ADP-ribosyl cyclase activity

Purified proteins (mBST-1, mCD38) were incubated with 0.5 mM β-NAD at 37 °C for 1 h in 20 mM Tris–HCl buffer (pH 7.2) with 0.1% Triton-X 100. Samples were treated with 0.6 M perchloric acid (PCA), and precipitates were removed by centrifugation. PCA was removed by mixing the aqueous sample with 3 parts 2 M KHCO_3_. After centrifugation at 15,000×*g* for 10 min, the aqueous layer was collected and neutralized with 20 mM sodium phosphate (pH 8.0). The enzyme product, cADPR, was measured by modification of the cycling method described previously [68]. To remove all contaminating nucleotides, the samples were incubated overnight with the following hydrolytic enzymes at 37 °C: 0.44 units/ml nucleotide pyrophosphatase, 12.5 units/ml alkaline phosphatase, 0.0625 units/ml NAD glycohydrolase, and 2.5 mM MgCl_2_ in 20 mM sodium phosphate buffer (pH 8.0). Enzymes were removed by filtration using Centricon 3 filters. To convert cADPR to β-NAD^+^, the samples (0.1 ml/tube) were incubated with 50 μl of a cycling reagent containing 0.3 μg/ml Aplysia ARC, 30 mM nicotinamide, and 100 mM sodium phosphate (pH 8.0) at room temperature for 30 min. The samples were further incubated with the cycling reagent (0.1 ml) containing 2% ethanol, 100 μg/ml alcohol dehydrogenase, 20 μM resazurin, 10 μg/ml diaphorase, 10 μM riboflavin 5′-phosphate, 10 mM nicotinamide, 0.1 mg/ml bovine serum albumin (BSA), and 100 mM sodium phosphate (pH 8.0) at room temperature for 2 h. An increase in resorufin fluorescence was measured at 544 nm excitation and 590 nm emission wavelengths using a fluorescence plate reader (Molecular Devices Corp., Spectra-Max GEMINI). Various known concentrations of cADPR were also included in the cycling reaction to generate a standard curve.

### Measurement of NAADP synthesis activity

NAADP synthesizing activity of the purified proteins (mBST-1, mCD38) was measured using a combination of the base-exchange reaction and the cycling method as described previously with modifications [69, 70]. The enzyme product, NAADP, was measured following incubation of the proteins with 0.5 mM NADP and 10 mM nicotinic acid at 37 °C for 1 h in 50 mM sodium acetate-acetic acid buffer (pH 4.5). Samples were treated with 0.6 M PCA, and precipitates were removed by centrifugation. PCA was removed by mixing the aqueous sample with 3 parts 2 M KHCO_3_. After centrifugation at 15,000×*g* for 10 min, the aqueous layer was collected and neutralized with 20 mM sodium phosphate (pH 8.0). To remove all contaminating nucleotides, the samples were incubated overnight with the following hydrolytic enzymes at 37 °C: 2.5 units/ml apyrase, 0.125 units/ml NAD glycohydrolase, 2 mM MgCl_2_, 1 mM NaF, 0.1 mM PPi, and 0.16 mg/ml NMN-adenylyl transferase in 20 mM sodium phosphate buffer (pH 8.0). Enzymes were removed by filtration using Centricon 3 filters. After the hydrolytic treatment, alkaline phosphate (10 units/ml) was added to convert NAADP to NAAD, and the samples incubated overnight at 37 °C. The alkaline phosphate was removed by filtration using Centricon 3 filters. The samples were further incubated with the cycling reagent (30 μl) containing 2% ethanol, 100 μg/ml alcohol dehydrogenase, 20 μM resazurin, 10 μg/ml diaphorase, 10 μM riboflavin 5′-phosphate, 10 mM nicotinamide, 0.1 mg/ml BSA, and 100 mM sodium phosphate (pH 8.0) at room temperature for 4 h. An increase in the resorufin fluorescence was measured at 544 nm excitation and 590 nm emission wavelengths using a fluorescence plate reader (Spectra-Max GEMINI). Various known concentrations of NAADP were also included in the cycling reaction to generate a standard curve.

### Light–dark transition test

The light–dark transition was measured using the light–dark test chamber [42]. One chamber was brightly illuminated (250 lx), whereas the other was dark (2 lx). Mice were placed in the light arena and were allowed to move freely between the two chambers for 600 s. Each male mouse was placed in the center of the light chamber, and the mouse was allowed to run freely between the two chambers for 10 min. The trial was recorded for 10 min using the ANY-maze video system [66]. Latency to enter (defined by all four paws entering), time spent, entries, and distance traveled in the light chamber were recorded.

### Social avoidance test

Social avoidance behavior toward an unfamiliar C57BL/6 N mouse was measured in a two-stage social interaction test [42]. In the first 10-min test (target absent), the experimental mouse was allowed to freely explore a square-shaped arena (600 × 600 mm) containing a wire mesh cage (70 × 90 × 70 mm and bars spaced 5 mm apart) placed in the center of the arena. In the second 20-min test, the experimental mouse was reintroduced back into the arena with an unfamiliar C57BL/6 N male mouse in a wire mesh cage. Video tracking software (ANY-maze) was used to measure the amount of time the experimental mouse spent in the “interaction zone” (300 × 300 mm). Behavior was measured in mice at 20 min after an intraperitoneal injection of 0.3 ml of OT (100 ng/kg body weight) or PBS or without any treatment.

### Statistical analysis

Data were analyzed using one-way or two-way analysis of variance (ANOVA). *Post hoc* comparisons were conducted only when the main effect was statistically significant. *P*-values for multiple comparisons were adjusted using Bonferroni’s correction. All analyses were conducted using STATA data analysis and statistical software (StataCorp LP, College Station, TX, USA).

## Additional files

**Additional file 1.** CD38 and CD157 mRNA expression levels.

**Additional file 2.** cADPR and NAADP levels.

**Additional file 3.** Number of entries to the dark zone.

**Additional file 4.** Time spent mobile and immobile in the inside zone.

## Abbreviations

Arc: arcuate hypothalamic nucleus
ASD: autism spectrum disorder
BLA: basolateral amygdaloid nucleus anterior part
BST-1: bone marrow stromal cell antigen-1
*BST1*: a gene for BST-1
cADPR: cyclic ADP-ribose
CeL: central amygdaloid nucleus
GWASs: genome-wide association studies
KO: knockout
mBST-1: mouse CD157
mCD38: mouse CD38
MePV: medial amygdaloid nucleus: posteroventral part
MPOA: medial preoptic arear
NAADP: nicotinic acid adenine dinucleotide phosphate
OT: oxytocin
PD: Parkinson’s disease
S1: primary somatosensory cortex: barrel field
S1BF: prim somatosensory cortex
S2: secondary somatosensory cortex
SNP: single nucleotide polymorphism
